# Relationship between Body Adiposity Indices and Reversal of Metabolically Unhealthy Obesity 6 Months after Roux-en-Y Gastric Bypass

**DOI:** 10.3390/metabo14090502

**Published:** 2024-09-18

**Authors:** Mariana Luna, Silvia Pereira, Carlos Saboya, Andrea Ramalho

**Affiliations:** 1 Postgraduate Program in Internal Medicine, Medical School, Universidade Federal do Rio de Janeiro (UFRJ), Rio de Janeiro 21941-971, Brazil; 2 Micronutrients Research Center (NPqM), Institute of Nutrition, Universidade Federal do Rio de Janeiro (UFRJ), Rio de Janeiro 21941-916, Brazil; centromultidisciplinar@cmcbm.com.br (S.P.); cjsaboya@carlossaboya.com.br (C.S.); aramalho@nutricao.ufrj.br (A.R.); 3 Multidisciplinary Center for Bariatric and Metabolic Surgery, Rio de Janeiro 22280-020, Brazil; 4 Social Applied Nutrition Department, Institute of Nutrition, Universidade Federal do Rio de Janeiro (UFRJ), Rio de Janeiro 21941-916, Brazil

**Keywords:** metabolically healthy obesity, metabolically unhealthy obesity, insulin resistance, visceral adiposity, roux-en-Y gastric bypass, bariatric surgery, obesity

## Abstract

The factors determining the reversal of metabolically unhealthy obesity (MUO) to metabolically healthy obesity (MHO) after Roux-en-Y gastric bypass (RYGB) are not completely elucidated. The present study aims to evaluate body adiposity and distribution, through different indices, according to metabolic phenotypes before and 6 months after RYGB, and the relationship between these indices and transition from MUO to MHO. This study reports a prospective longitudinal study on adults with obesity who were evaluated before (T0) and 6 months (T1) after RYGB. Bodyweight, height, waist circumference (WC), BMI, waist-to-height ratio (WHR), total cholesterol (TC), HDL-c, LDL-c, triglycerides, insulin, glucose, HbA1c and HOMA-IR were evaluated. The visceral adiposity index (VAI), the conicity index (CI), the lipid accumulation product (LAP), CUN-BAE and body shape index (ABSI) were calculated. MUO was classified based on insulin resistance. MUO at T0 with transition to MHO at T1 formed the MHO-t group MHO and MUO at both T0 and T1 formed the MHO-m and MUO-m groups, respectively. At T0, 37.3% of the 62 individuals were classified as MHO and 62.7% as MUO. Individuals in the MUO-T0 group had higher blood glucose, HbA1c, HOMA-IR, insulin, TC and LDL-c compared to those in the MHO-T0 group. Both groups showed significant improvement in biochemical and body variables at T1. After RYGB, 89.2% of MUO-T0 became MHO (MHO-t). The MUO-m group presented higher HOMA-IR, insulin and VAI, compared to the MHO-m and MHO-t groups. CI and ABSI at T0 correlated with HOMA-IR at T1 in the MHO-t and MHO-m groups. CI and ABSI, indicators of visceral fat, are promising for predicting post-RYGB metabolic improvement. Additional studies are needed to confirm the sustainability of MUO reversion and its relationship with these indices.

## 1. Introduction

Obesity is a multifactorial, heterogeneous condition, associated with increased morbidity and mortality and is difficult to control, representing an important public health challenge worldwide [1]. Given the current interventions, bariatric surgery is capable of promoting substantial and sustainable weight loss, metabolic improvements and remission and/or mitigation of comorbidities. It is increasingly performed around the world and is currently recognized as the most effective alternative for obesity control [2,3,4,5,6,7,8].

Although clearly associated with the development of chronic non-communicable diseases, not all individuals with obesity present the expected metabolic changes, a condition known as “Metabolically Healthy Obesity” (MHO) [9,10,11,12,13,14]. This phenotype, although apparently protective, appears to be transient and may evolve into “Metabolically Unhealthy Obesity” (MUO) in 5 to 10 years, increasing the risk of cardiometabolic complications [15,16,17].

The transition from MUO to MHO is also possible, through appropriate interventions. Bariatric surgery, for example, results in reversal of MUO in 80–90% of cases, improving health condition regardless of body weight [18,19,20]. However, the factors associated with this transition are not well understood. Furthermore, the effectiveness and applicability of the surgery in individuals with MHO is still questioned in the literature.

Research into the metabolic differences between MHO and MUO has recently evolved, and the literature highlights the importance of body composition, especially adiposity and its distribution. Regardless of BMI, excessive visceral fat accumulation is associated with greater inflammation and insulin resistance (IR), representing an increased cardiovascular risk [11,21]. MHO individuals accumulate more subcutaneous and less visceral fat, which preserves insulin sensitivity and reduces cardiometabolic risk [11,12,15,21,22].

Therefore, evaluating fat distribution is essential for a more complete approach to obesity management, as it supports the development of effective strategies and evaluates their effectiveness. Since the gold standard methods used for this purpose, such as computed tomography (CT), dual X-ray absorptiometry (DXA) and magnetic resonance imaging (MRI), are not viable in clinical practice and common tools, such as BMI, waist circumference (WC) and waist-to-height ratio (WHtR), do not distinguish between subcutaneous and visceral fat, studies have recommended the use of indices of body adiposity that are easy to apply, low cost and well correlated with gold standard methods, such as the visceral adiposity index (VAI), the lipid accumulation product (LAP), adiposity estimator—Clinical University of Navarra (CUN-BAE), the conicity index (CI) and a body shape index (ABSI) [23,24,25,26,27,28].

However, data on the use of these indices in individuals undergoing bariatric surgery are scarce, especially in relation to their association with metabolic phenotypes and their transition. The present study aims to evaluate body adiposity and its distribution through different indices (BMI, WC, WHtR, VAI, LAP, CUN-BAE, CI and ABSI), as well as the metabolic phenotypes of obesity before and 6 months after RYGB. In addition, it aims to investigate the relationship between these indices pre-surgery and the transition from MUO to MHO after surgery.

## 2. Materials and Methods

### 2.1. Study Design and Population

This was a prospective longitudinal study, involving individuals undergoing RYGB, who were followed up at a multidisciplinary center specialized in obesity control, the “Multidisciplinary Center for Bariatric and Metabolic Surgery”, in Rio de Janeiro, Brazil. They were evaluated before (T0) and 6 months (T1) after surgery. The sample was selected for convenience, where all patients seen between January 2018 and September 2020 were invited to participate in the study.

Inclusion criteria were the following: age between 20 and <60 years, both men and women, with indication for RYGB. The exclusion criteria were the following: previous malabsorptive and restrictive surgeries, intestinal malabsorptive syndromes, neoplasms, use of drugs for weight loss, alcohol consumption greater than 20 g/day for women and 40 g/day for men, pregnancy or lactation, renal failure (defined by estimated glomerular filtration rate <60 mL/min/1.73 m^2^), liver diseases (except non-alcoholic fatty liver disease), acute or chronic infections, high serum calcium levels, endocrinopathies (hyperparathyroidism, hypothyroidism, hypercortisolemia) and use of anticonvulsant medications.

The present study was approved by the Research Ethics Committee of Hospital Clementino Fraga Filho/UFRJ (protocol number 011/10). The inclusion of participants was carried out with formal authorization, including the signing of a free and informed consent form.

All patients underwent RYGB via laparoscopy and were followed up by the clinic’s multidisciplinary team.

### 2.2. Data Collection

Sociodemographic (sex and age) and body (weight, height and waist circumference (WC)) data were collected in a previously scheduled consultation, part of the clinic’s routine care, both at T0 and T1, by a single trained evaluator.

WC was measured in accordance with the WHO recommendations. The cutoff points adopted were ≥94 cm for men and ≥80 cm for women [29]. The waist-to-height ratio (WHtR) was calculated (WC/Height), with a cutoff point of 0.5. BMI was calculated (weight (kg)/height (m^2^)) and categorized according to WHO, 2000 [29].

The percentage (%) of weight loss (%WL) and percentage (%) of excess weight loss (%EWL) after surgery were calculated at T1. For %WL, the following formula was used: [(weight T0 (Kg)) − (weight T1 (Kg))]/[(weight T0 (Kg))] × 100; and for %EWL: [(BMI T0) − (BMI T1)]/[(BMI T0) − (BMI 25)].

Blood was collected after a 12-h fast, both at T0 and T1, to quantify total cholesterol (TC), HDL-c, LDL-c (by Friedwald formula), triglycerides (TGs), fasting blood glucose, glycated hemoglobin (HbA1 c) and insulin. To assess insulin resistance, the homoeostasis model assessment for insulin resistance (HOMA-IR) was calculated [30].

To evaluate the adequacy of biochemical parameters, the following cutoff points were applied: CT < 190 mg/dL, LDL-c < 130 mg/dL, HDL-c > 40 mg/dL, TG < 150 mg/dL [31], glucose < 100 mg/dL, glycated hemoglobin < 5.7%, insulin 2.0–17 mcU/mL and HOMA-IR ≥ 2.5 [30,32].

To evaluate body adiposity from the anthropometric and biochemical variables obtained, VAI, LAP, CUN-BAE, CI and ABSI were calculated for T0 and T1, using the following formulas:

- VAI [23]:

Men: $\left(\frac{\mathrm{WC}\;}{39.68+\left(1.88\times \mathrm{BMI}\right)}\right)\times \left(\frac{\mathrm{TG}}{1.03}\right)\times \left(\frac{1.31}{\mathrm{HDL}}\right)$;

Women: $\left(\frac{\mathrm{WC}\;}{36.58+\left(1.88\times \mathrm{BMI}\right)}\right)\times \left(\frac{\mathrm{TG}}{0.81}\right)\times \left(\frac{1.52}{\mathrm{HDL}}\right)$

- LAP [24]:

Men: $\left(\mathrm{WC}-65\right)\times \mathrm{TG}$;

Women: $\left(\mathrm{WC}-58\right)\times \mathrm{TG}$

- CUN-BAE [25]:

$\begin{array}{cc} -44.98+(0.503 & \times \;\mathrm{age})+\left(10.689\times \mathrm{sex}\right)+\left(3.172\times \mathrm{BMI}\right)-\left(0.026\times \mathrm{BMI}\right)\\ & +\left(0.181\times \;\mathrm{BMI}\times \;\mathrm{sex}\right)-\left(0.02\times \;\mathrm{BMI}\times \mathrm{age}\right)-\left(0.005\times \mathrm{BMI}\times \mathrm{sex}\right)\\ & +\left(0.00021\times \mathrm{BMI}\times \mathrm{Age}\right) \end{array}$

Sex: men = 0; women = 1

- CI [26]:

$\frac{\mathrm{WC}\;\left(m\right)}{\left(0.109\times \sqrt{\frac{\mathrm{Weight}\left(\mathrm{kg}\right)}{\mathrm{Height}\left(m\right)}}\right)}$

- ABSI [25]:

$\frac{\mathrm{WC}\;\left(m\right)}{{\mathrm{BMI}}^{2/3}\times {\mathrm{Height}}^{1/2}}$

VAI also presents a cutoff point for inadequacy, proposed by its creators. Thus, these points were adopted to identify the presence of dysfunctional visceral adipose tissue [33].

### 2.3. Metabolic Phenotypes

HOMA-IR was used as the criterion to classify metabolic phenotypes [34,35,36,37]. Individuals with HOMA-IR ≥ 2.5 at T0 were classified with MUO (MUO-T0), and those with HOMA-IR < 2.5, with MHO (MHO-T0).

In order to evaluate possible factors associated with the transition from MUO to MHO after RYGB, MUO-T0 individuals who became metabolically healthy at T1 (HOMA-IR < 2.5) were subdivided into the MHO-t group. Those classified as MHO at both T0 and T1 were subdivided into the MHO-m group, and those who remained MUO at both times, into the MUO-m group.

### 2.4. Statistical Analyses

Data distribution was evaluated using the Kolmogorov–Smirnov test. From this analysis, it was observed that data had a non-normal distribution, and therefore, the analyses adopted were non-parametric. Continuous variables were expressed as mean ± standard deviation, and categorical variables were expressed as percentages.

To evaluate the difference between the independent groups, the Mann–Whitney test was used for numerical variables and Chi-square or Fisher’s exact tests for nominal categorical variables. For the intra-group comparison of numerical variables between T0 and T1, the Wilcoxon test was used and, for nominal categorical variables, the Mc Nemar test. The Spearman correlation test was used to evaluate the association between adiposity indices at T0 and the variables involved in characterizing the phenotype (insulin and HOMA-IR) at T1.

## 3. Results

### 3.1. Sample Characterization and Post-Surgical Outcomes According to the Metabolic Obesity Phenotype

A total of 62 individuals were included in this study, with a mean age of 42.8 ± 11.1 years, and 80% were female. The MHO-T0 and MUO-T0 groups comprised 37.3% (*n* = 22) and 62.7% (*n* = 37) of the sample, respectively. A proportion of 81.8% (*n* = 18) of the MHO-T0 and 78.4% (*n* = 29) of the MUO-T0 individuals were female, with no significant difference between groups (*p* > 0.05). There was also no significant difference regarding age (MHO-T0: 44.0 ± 11.3 years vs. MUO-T0: 42 ± 11.3 years, *p* = 0.456). After surgery, 91.7% (*n* = 55) of individuals were classified as MHO and 8.3% (*n* = 5) as MUO.

Before surgery, the MUO-T0 group had higher means of fasting blood glucose, HbA1c, HOMA-IR, insulin, TC and LDL-c. After RYGB, only TC remained higher in this group, in addition to higher CI, compared to MHO-T0. Both phenotypes showed significant improvement in biochemical and body variables at 6 months post-surgery, with the exception of HDL (Table 1).

Considering the modification in the biochemical and body variables after surgery, the MUO-T0 group showed a greater reduction in HOMA-IR and insulin levels and a smaller variation in ABSI compared to the MHO-T0 group. There was no difference in relation to weight loss or changes in other variables (Table 2).

Regarding metabolic changes, there was a lower prevalence of high blood glucose, HbA1c, TC and LDL-c in the MHO-T0 group compared to MUO-T0. When comparing T1 to T0, all the metabolic changes improved in the MUO-T0 group. For MHO-T0, there were no statistical difference between T0 and T1, except for the prevalence of high TC. For the MUO-T0 group, there was a significant reduction in the prevalence of all changes 6 months after RYGB, reaching percentages similar to those in the MHO-T0 group after surgery (Table 3).

### 3.2. Transition to Metabolically Healthy Obesity Phenotype 6 Months after RYGB and Its Association with Body Adiposity Indices

A proportion of 95.5% (*n* = 21) of the individuals classified as MHO-T0 remained in this phenotype at T1, forming the MHO-m subgroup. In relation to MUO-T0, 89.2% (*n* = 33) had improved metabolic health and transitioned to MHO at T1, forming the MHO-t subgroup. On the other hand, 10.8% (*n* = 4) maintained the unhealthy phenotype, constituting the MUO-m subgroup.

When comparing the body and biochemical variables of the three subgroups, the MUO-m individuals showed higher means of HOMA-IR, insulin and VAI compared to the two healthy groups (Table 4). When comparing MHO-t and MHO-m, the means were similar for almost all variables evaluated, with the exception of VAI and LAP, which were higher in the MHO-t group.

To evaluate the possible influence of adiposity indices on phenotype transition after surgery, the correlations between these pre-surgical indices and the post-surgical HOMA-IR, the criterion used to classify metabolic phenotype in the present study, were evaluated. In the MHO-t and MHO-m subgroups, pre-surgical CI and ABSI were correlated with postsurgical HOMA-IR. These results suggest that CI and ABSI are potential predictors of insulin resistance in specific contexts, aligning with this study’s objective of identifying markers of metabolic success after surgery.

**Figure 1:**
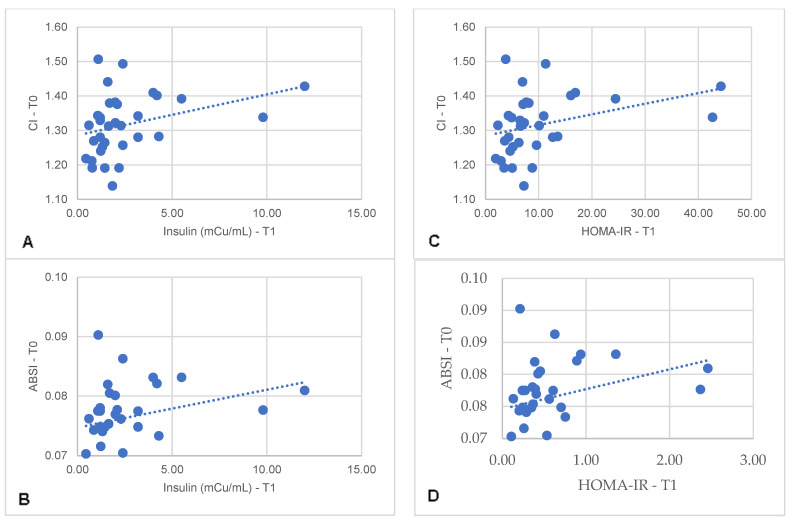
Correlation between serum insulin concentrations 6 months after Roux-en-Y gastric bypass with pre-surgical CI (**A**) and ABSI (**B**) and correlation between HOMA-IR 6 months after surgery with pre-surgical CI (**C**) and ABSI (**D**). CI: conicity index; ABSI: a body shape index.

**Figure 2:**
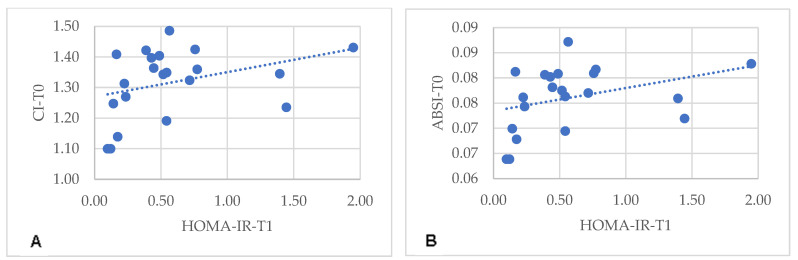
Correlation between HOMA-IR 6 months after Roux-en-Y gastric bypass with pre-surgical CI (**A**) and ABSI (**B**). CI: conicity index; ABSI: a body shape index.

Specifically, in the MHO-t subgroup, CI and ABSI at T0 correlated with insulin (r = 0.389, *p* = 0.025; r = 0.362, *p* = 0.038) and with HOMA-IR at T1 (r = 0.440, *p* = 0.010; r = 0.425, *p* = 0.014) (Figure 1). Similarly, in the MHO-m subgroup, CI and ABSI at T0 correlated with HOMA-IR at T1 (r = 0.444, *p* = 0.044; r = 0.481, *p* = 0.027) (Figure 2). In both subgroups, there were no significant correlations between HOMA-IR at T1 with other body variables at T0, such as BMI (r = −0.053, *p* = 0.765), WC (r = 0.355, *p* = 0.390), WHtR (r =0.259, *p* = 0.139), LAP (r = −0.074, *p* = 0.687), VAI (r = −0.134, *p* = 0.464) or CUN-BAE (r = −0.243, *p* = 0.165). These findings indicate that, in the MHO-t and MHO-m subgroups, CI and ABSI are more consistent indicators of post-surgical IR compared to other body variables, reinforcing the relevance of these indices in predicting positive metabolic outcomes.

On the other hand, in the MUO-m subgroup, no significant correlation was observed between the body variables at T0 and post-surgical HOMA-IR (BMI: r = 0.100, *p* = 0.800; WC: r = 0.400, *p* = 0.600; WHtR: r = 0.800, *p* = 0.200; LAP: r = −0.400, *p* = 0.600; CI: r = −0.400, *p* = 0.600; ABSI: r = −0.400, *p* = 0.600; VAI: r = 0.500; CUN-BAE: r = 0.100, *p* = 0.800). This result suggests that, in this specific group, the body variables analyzed were not good indicators of IR after surgery, highlighting the need to explore other factors that may influence the results in this subgroup.

## 4. Discussion

In the present study, six months after RYGB, there was a reversal from MUO to MHO in 89% of cases, demonstrating the effectiveness of the intervention not only in weight loss but also in early metabolic improvement, which contributes to the reduction of morbidity and mortality in obesity. Understanding the factors associated with metabolic health transition is essential to reducing the obesity-related risks and enhance the benefits of RYGB in the short, medium and long term.

This pioneering study evaluated eight body adiposity indices (BMI, WC, WHtR, VAI, CI, LAP, ABSI and CUN-BAE) in relation to metabolic phenotypes and their changes six months after RYGB. The results highlight the importance of pre-surgical CI and ABSI, which were positively correlated with post-surgical HOMA-IR in individuals who changed from MUO to MHO and also in those who remained MHO, suggesting their potential as predictors of the reversal of unhealthy phenotype and maintenance of the healthy phenotype after surgery, thus aligning with this study’s objective of identifying indicators of metabolic success after surgery.

IR, assessed by HOMA-IR, is fundamental for the development and worsening of metabolic changes in obesity, and preserved insulin sensitivity is a characteristic of MHO individuals. HOMA-IR is an easy-to-apply and useful tool for the early detection of cardiometabolic risk, being more effective than traditional criteria for classifying phenotypes such as NCEP-ATP III or IDF criteria [20,21,34,35,36,37,38].

The maintenance of insulin sensitivity is influenced by body fat distribution. Greater visceral adiposity in relation to subcutaneous fat indicates greater adipose tissue dysfunction, release of pro-inflammatory cytokines and increased IR [9,21,39,40,41]. Thus, within the same BMI range, it is possible to observe different health impairment degrees, depending on fat distribution [21,40,42,43]. MHO individuals tend to accumulate more subcutaneous fat, while MUO individuals tend to accumulate more visceral fat [11,21].

Assessing visceral fat is essential to correctly evaluate health in obesity and to analyze the effectiveness of strategies to control the disease. However, gold standard methods, such as computed tomography (CT), dual X-ray absorptiometry (DXA) and magnetic resonance imaging (MRI), are expensive and present risks, making their routine application unfeasible. On the other hand, the body adiposity indices studied (CI, ABSI, VAI, LAP and CUN-BAE) show good correlation with these methods, offering accurate, low-cost and easy-to-obtain alternatives to translate information about adiposity and metabolic health [27,44,45,46,47,48].

ABSI is associated with greater health risk and is more effective than BMI or WC in predicting all-cause mortality [46,49,50]. This index reflects body shape and central fat accumulation with little influence from BMI, based on epidemiological statistics [28,47]. CI, in turn, is calculated from a biophysical perspective, assuming that the human body has a cylindrical shape [26]. The greater the central fat accumulation, the greater the CI, which strongly correlates with the visceral fat area measured by CT [48].

A relevant aspect is that CI and ABSI are interrelated, showing a greater correlation between them, compared to any other adiposity indices evaluated, as observed in a study with more than 62,000 individuals [47]. This indicates that, despite their distinct natures (epidemiological and biophysical, respectively), these indices are significantly similar.

In the present study, unlike ABSI and CI, pre-surgical BMI did not correlate with post-surgical HOMA-IR, highlighting that body fat distribution is more influent than initial total body mass in obesity metabolic improvement. Although promising, the application of adiposity indices such as ABSI and CI in individuals undergoing RYGB is scarce in the literature, especially in relation to obesity metabolic phenotypes. However, previous studies using gold standard methods also indicate that changes in fat depots are more associated with metabolic improvement than weight loss or BMI reduction [4,18,51].

In individuals evaluated before and 1 year after RYGB, visceral fat, assessed by MRI, showed a significant association with arterial pulse wave, an important predictor of cardiovascular health. This association was not observed when considering BMI or WC [51]. In the same post-surgical period, greater pre-surgical visceral adiposity, also assessed by MRI, was associated with a higher DM2 remission rate [4].

Regarding phenotype transition, the present results are in line with previous studies using gold standard methods. In a study that applied CT to individuals before and one year after sleeve or RYGB, those who transitioned from MUO to MHO showed a greater reduction in ectopic fat, but not in subcutaneous fat, when compared to those who remained MUO, and there was no relationship between initial BMI and this transition [18]. In the present study, using VAI as an indicator of visceral adiposity and adipose tissue dysfunction, it was observed that individuals who maintained the unhealthy phenotype had a higher mean of this index, compared to the two groups classified as healthy 6 months after surgery (MHO-t and MHO-m). However, this difference was not observed in relation to BMI, WC or WHtR.

In individuals with metabolic syndrome undergoing different surgical techniques (RYGB, sleeve and duodenal switch), evaluated by CT before and 6 months after surgery, there was no difference between BMI, total body fat mass or weight loss when comparing the group of those who transitioned to a healthy state with those who did not. The only significant difference was the visceral fat content at baseline. The individuals who maintained MUO had greater pre-surgical visceral fat [52].

Adding previous evidence to the present findings, the limitation of BMI in predicting health improvements after RYGB is reinforced, highlighting the importance of fat distribution and the usefulness of body adiposity indices, especially for those classified as MUO before surgery.

It is important to highlight that, in addition to visceral fat, ABSI is inversely related to fat-free mass, especially musculoskeletal, assessed by gold standard methods such as DXA, being an important predictor of sarcopenic obesity. [44,45,50,53]. Fat-free mass is crucial for metabolic health, and its reduction is associated with greater cardiovascular risk in individuals with obesity, regardless of metabolic phenotype [54,55]. Sarcopenia is related to IR, increasing the risk of cardiovascular and all-cause mortality in obesity [56,57].

In a prospective longitudinal study with more than 9000 individuals, those classified as MHO and in the highest ABSI quartiles had a higher risk of cardiovascular disease compared to those of adequate weight and healthy, but in the first ABSI quartile. However, those with MHO and in the first ABSI quartile presented a similar risk to those with adequate weight, despite the presence of obesity, defined by BMI. Lower ABSI was related to a higher fat-free mass content, exerting a protective effect on health [56].

In a study with approximately 25,000 individuals, those with MHO were at increased risk of cardiovascular disease in the presence of sarcopenia. In the absence of sarcopenia, there was no significant difference in risk compared to healthy individuals of adequate weight, highlighting the importance of musculoskeletal mass for cardiometabolic health [50]. In the first six months post-RYGB, a highly catabolic period, there is greater loss of fat and fat-free mass [58]. Therefore, ABSI application can be extremely useful in post-surgical monitoring for the early detection of both visceral adiposity and loss of musculoskeletal mass, providing a more complete assessment of patients’ health.

In the present study, before surgery, MUO-T0 individuals presented higher means of HbA1c, insulin, TC and LDL-c and higher prevalence of metabolic changes compared to MHO-T0. After surgery, there was a reversal of metabolic changes, with MUO-T0 reaching a profile similar to MHO-T0. The improvement in biochemical variables and adiposity indices was similar in both groups, indicating that the intervention is beneficial even for individuals classified as healthy before surgery, addressing questions raised in the scientific literature.

MHO is not a stable condition and can evolve into MUO in about 60% of cases within 10 years [59]. Visceral and ectopic fat are independent predictors of this transition. MHO individuals with greater initial visceral/ectopic adiposity are more likely to have long-term metabolic worsening and progression to MUO [15,16,59]. In the present study, visceral fat reduction, indicated by the reduction in all assessed adiposity indices, together with the improvement in important variables for cardiovascular risk, suggests that MHO can be prolonged by bariatric surgery, reducing the likelihood of progression to MUO in the long term. These findings reinforce the usefulness of surgery even in individuals initially classified as MHO, contributing positively to debates about its effectiveness across different health profiles [3,60].

The present study has some limitations, such as its small sample size and a follow-up period of only 6 months, preventing the extrapolation of the results to long-term observations. However, it is important to highlight the relevance of this period, as it coincides with the peak in visceral adiposity reduction, which tends to stabilize later [18,61]. A strength of this study lies in its pioneering nature in evaluating eight different indices of body adiposity in relation to metabolic phenotypes and their changes six months after RYGB. This more comprehensive approach provides an important foundation for clinical practice and future research.

Given the present findings, the usefulness of CI and ABSI as potential predictors of a favorable metabolic transition 6 months after RYGB stands out. Both indices are accurate and accessible alternatives for visceral fat and metabolic health assessment, being promising as tools for patient screening, the selection of individualized strategies and the prediction of post-surgical outcomes.

We reinforce the need for additional studies with larger samples and prolonged follow-up to investigate the sustainability of MUO reversal and its relationship with adiposity indices, aiming to enrich post-surgical follow-up.

## Figures and Tables

**Table 1:** Biochemical and body variables of individuals undergoing Roux-en-Y gastric bypass, before and 6 months after surgery, according to their pre-surgical metabolic phenotype (mean ± SD).

VARIABLES	T0		p *	T1		p *	T0 × T1 *p* ^+^	
	MHO-T0 (*n* = 22)	MUO-T0 (*n* = 37)		MHO-T0 (*n* = 22)	MUO-T0 (*n* = 37)		MHO	MUO
BMI (Kg/m^2^)	42.1 ± 4.1	41.3 ± 3.9	0.451	32.9 ± 4.1	31.2 ± 4.1	0.771	1 × 10^−4^	1 × 10^−4^
WC (cm)	121.4 ± 14.6	118.6 ± 10.1	0.389	98.1 ± 9.8	97.7 ± 8.8	0.306	1 × 10^−4^	1 × 10^−4^
WtHR	0.71 ± 0.07	0.7 ± 0.1	0.300	0.6 ± 0.1	0.6 ± 0.1	0.393	1 × 10^−4^	1 × 10^−4^
Glucose (mg/dL)	90.5 ± 7.7	104.7 ± 23.5	0.684	85.7 ± 9.8	91.1 ± 10.2	0.527	0.026	1 × 10^−4^
HbA1c (%)	5.2 ± 0.4	6.9 ± 7.3	0.003	3.5 ± 1.2	9.9 ± 4.6	0.561	1 × 10^−5^	1 × 10^−4^
HOMA-IR	1.7 ± 0.5	4.6 ± 1.6	0.013	0.5 ± 0.41	0.9 ± 1.1	0.804	0.001	1 × 10^−4^
Insulin (mCu/mL)	7.6 ± 1.9	19.6 ± 7.6	1 × 10^−4^	2.7 ± 2.3	3.9 ± 4.6	0.974	0.001	1 × 10^−4^
TC (mg/dL)	184.7 ± 41.8	219.9 ± 63.2	1 × 10^−5^	123.1 ± 34.9	135.1 ± 42.1	1 × 10^−4^	1 × 10^−4^	1 × 10^−4^
LDL-c (mg/dL)	108.2 ± 30.5	133.1 ± 53.9	0.026	84.0 ± 15.6	95.6 ± 34.9	0.091	0.001	1 × 10^−4^
HDL-c (mg/dL)	43.5 ± 19.9	43.5 ± 9.8	0.080	47.5 ± 17.1	46.2 ± 11.7	0.105	0.164	0.100
TG (mg/dL)	139.4 ± 87.8	155.1 ± 63.6	0.796	113.6 ± 78.7	120.2 ± 48.2	0.088	0.001	1 × 10^−5^
CRP (mg/dL)	0.8 ± 0.8	0.9 ± 0.8	0.085	0.7 ± 0.5	0.7 ± 0.5	0.282	0.050	0.001
VAI	2.8 ± 1.8	3.0 ± 1.5	0.632	1.6 ± 1.0	2.2 ± 1.2	0.823	0.026	1 × 10^−4^
CI	1.3 ± 0.1	1.3 ± 0.1	0.294	1.2 ± 0.1	1.3 ± 0.1	0.023	1 × 10^−4^	1 × 10^−4^
ABSI	0.08 ± 0.001	0.08 ± 0.001	0.638	0.07 ± 0.001	0.08 ± 0.001	0.389	0.009	0.922
CUN-BAE	51.4 ± 4.4	50.3 ± 5.6	0.913	43.3 ± 6.7	40.7 ± 8.2	0.271	1 × 10^−4^	1 × 10^−4^
LAP	192.3 ± 126.6	206.2 ± 84.5	0.638	100.85 ± 38.31	131.9 ± 52.8	0.077	1 × 10^−4^	1 × 10^−5^

SD: standard deviation; T0: baseline; T1: 6 months post-surgery; BMI: body mass index; WC: waist circumference; WtHR: waist-to-hip ratio; TC: total cholesterol; LDL-c: low density lipoprotein; HDL-c: high density lipoprotein; TG: triglycerides; CRP: c-reactive protein; VAI: visceral adiposity index; CI: conicity index; ABSI: a body shape index; CUN-BAE: body adiposity estimator—Clinica Universidad de Navarra; LAP: lipid accumulation product. * Mann–Whitney test; ^+^ Wilcoxon test.

**Table 2:** Variation in biochemical and body variables of individuals undergoing Roux-en-Y gastric bypass, 6 months after surgery, according to their pre-surgical metabolic phenotype (mean ± SD).

Δ VARIABLES	MHO-T0 (*n* = 22)	MUO-T0 (*n* = 37)	*p* *
Weight (Kg)	−36.68 ± 10.30	−27.88 ± 10.00	0.695
%WL	54.98 ± 18.85	64.35 ± 23.26	0.227
%EWL	21.69 ± 7.08	24.55 ± 7.54	0.113
BMI (Kg/m^2^)	−9.16 ± 3.12	−10.15 ± 3.15	0.125
WC (cm)	−23.27 ± 8.96	−20.86 ± 7.24	0.237
WtHR	−0.14 ± 0.05	−0.12 ± 0.05	0.397
Glucose (mg/dL)	−4.77 ± 8.82	−13.51 ± 20.46	0.057
HbA1c (%)	−1.66 ± 1.18	−3.05 ± 7.16	0.433
HOMA-IR	−1.14 ± 0.67	−4.17 ± 2.14	1 × 10^−4^
Insulin (mCu/mL)	−4.98 ± 2.87	−15.73 ± 7.31	1 × 10^−4^
TC (mg/dL)	−61.58 ± 40.39	−84.78 ± 59.87	0.207
LDL-c (mg/dL)	−24.22 ± 29.98	−37.49 ± 43.48	0.173
HDL-c (mg/dL)	4.04 ± 16.62	2.70 ± 12.39	0.350
TG (mg/dL)	−25.86 ± 37.37	−34.77 ± 48.98	0.476
CRP (md/dL)	−0.15 ± 0.35	−0.21 ± 0.42	0.493
VAI	−1.12 ± 1.09	−0.69 ± 1.1	0.802
CI	−0.11 ± 0.08	−0.06 ± 0.09	0.074
ABSI	−0.003 ± 0.01	−0.000 ± 0.01	0.027
CUN-BAE	−8.07 ± 3.50	−9.60 ± 4.06	0.106
LAP	−60.05 ± 57.20	−74.38 ± 69.50	0.500

SD: standard deviation; T0: baseline; Δ: variation; %WL: %weight loss; %EWL: % excess weight loss; BMI: body mass index; WC: waist circumference; WtHR: waist-to-hip ratio; TC: total cholesterol; LDL-c: low density lipoprotein; HDL-c: high density lipoprotein; TG: triglycerides; CRP: c-reactive protein; VAI: visceral adiposity index; CI: conicity index; ABSI: a body shape index; CUN-BAE: body adiposity estimator—Clinica Universidad de Navarra; LAP: lipid accumulation product. * Mann–Whitney test.

**Table 3:** Prevalence of metabolic changes in individuals undergoing Roux-en-Y gastric bypass, before and 6 months after surgery, according to their pre-surgical metabolic phenotype (% (*n*)).

METABOLIC CHANGES	T0		*p* *	T1		*p* *	*p* MHO T0 vs. T1 ^+^	*p* MUO T0 vs. T1 ^+^
	MHO-T0 (*n* = 22)	MUO-T0 (*n* = 37)		MHO-T0 (*n* = 22)	MUO-T0 (*n* = 37)			
High glucose	13.6% (3)	51.4% (19)	0.004	4.5% (1)	16.2% (6)	0.240	0.625	1 × 10^−4^
High HbA1c	0.0% (0)	24.3% (9)	0.012	0.0% (0)	0.0% (0)	-	-	0.004
High TC	27.3% (6)	62.2% (23)	0.010	0.0% (0)	2.7% (1)	0.627	0.031	1 × 10^−4^
High LDL-c	18.2% (4)	45.9% (17)	0.031	0.0% (0)	13.5% (5)	0.146	0.125	0.002
Low HDL-c	40.9% (9)	45.9% (17)	0.706	22.7% (5)	21.6% (8)	0.921	0.344	0.049
High TG	36.4% (8)	51.4% (19)	0.264	13.6% (3)	27.0% (10)	0.230	0.063	0.004

T0: baseline; T1: 6 months post-surgery; TC: total cholesterol; LDL-c: low density lipoprotein; HDL-c: high density lipoprotein; TG: triglycerides/* Chi-square test or Fisher’s exact test (comparison of nominal qualitative variables between independent groups); ^+^ McNemar test (comparison of nominal qualitative variables between dependent groups).

**Table 4:** Biochemical and body variables of individuals 6 months after Roux-en-Y gastric bypass according to metabolic phenotype transition (Mean ± SD).

VARIABLES	MHO-t (*n* = 34)	MHO-m (*n* = 21)	MUO-m (*n* = 4)	*p*
Age (Years)	42.03 ± 11.26 ^a^	43.43 ± 11.25 ^a^	42.75 ± 11.35 ^a^	0.828
BMI (Kg/m^2^)	31.07 ± 4.30 ^a^	32.83 ± 4.18 ^a^	32.67 ± 2.45 ^a^	0.357
WC (cm)	97.10 ± 9.97 ^a^	98.05 ± 10.06 ^a^	99.50 ± 5.00 ^a^	0.843
WtHR	0.59 ± 0.05 ^a^	0.58 ± 0.05 ^a^	0.63 ± 0.04 ^a^	0.300
Glucose (mg/dL)	90.68 ± 10.23 ^a^	85.47 ± 9.97 ^a^	94.75 ± 9.22 ^a^	0.153
HbA1c (%)	3.94 ± 1.05 ^a^	3.61 ± 1.22 ^a^	2.77 ± 0.62 ^a^	0.124
HOMA-IR	0.55 ± 0.54 ^a^	0.57 ± 0.48 ^a^	3.52 ± 0.70 ^b^	0.004
Insulin (mCu/mL)	2.47 ± 2.45 ^a^	2.70 ± 2.23 ^a^	14.95 ± 1.55 ^b^	0.003
TC (mg/dL)	140.71 ± 41.12 ^a^	123.25 ± 35.76 ^a^	92.50 ± 5.32 ^b^	0.031
LDL-c (mg/dL)	95.32 ± 35.00 ^a^	83.71 ± 15.91 ^a^	97.00 ± 34.47 ^a^	0.503
HDL-c (mg/dL)	46.52 ± 11.97 ^a^	47.28 ± 17.43 ^a^	45.50 ± 9.75 ^a^	0.470
TG (mg/dL)	125.94 ± 58.74 ^a^	103.67 ± 65.20 ^a^	122.00 ± 57.50 ^a^	0.125
CRP (mg/dL)	0.74 ± 0.58 ^a^	0.64 ± 0.51 ^a^	0.53 ± 0.33 ^a^	0.865
VAI	2.16 ± 1.11 ^a^	1.41 ± 0.62 ^b^	3.33 ± 2.00 ^c^	0.010
CI	1.25 ± 0.11 ^a^	1.20 ± 0.10 ^a^	1.27 ± 0.04 ^a^	0.322
ABSI	0.08 ± 0.01 ^a^	0.07 ± 0.01 ^a^	0.08 ± 0.00 ^a^	0.227
CUN-BAE	40.70 ± 8.45 ^a^	43.07 ± 6.77 ^a^	42.50 ± 5.03 ^a^	0.619
LAP	131.50 ± 53.14 ^a^	100.85 ± 38.31 ^b^	135.92 ± 60.05 ^a^	0.040

Kruskal–Wallis. Different superscript letters indicate significant statistical differences in the pairwise comparisons according to the Bonferroni test (*p* < 0.05); SD: standard deviation; MHO-t: transition to metabolically healthy obesity post-RYGB; MHO-m: maintenance of metabolically healthy obesity after RYGB; MUO-m: maintenance of metabolically unhealthy obesity after RYGB; BMI: body mass index; WC: waist circumference; WtHR: waist-to-hip ratio; TC: total cholesterol; LDL-c: low density lipoprotein; HDL-c: high density lipoprotein; TG: triglycerides; CRP: c-reactive protein; VAI: visceral adiposity index; CI: conicity index; ABSI: a body shape index; CUN-BAE: body adiposity estimator—Clinica Universidad de Navarra; LAP: lipid accumulation product.

## Data Availability

The data presented in this study are available on request from the corresponding author due to privacy.
